# Case of Hydrophobia

**Published:** 1864-06

**Authors:** R. S. Addison

**Affiliations:** Chicago, Ill.


ARTICLE XXVI.

### CASE OF HYDROPHOBIA.

BY R. S. ADDISON, M.D., Chicago, Ill.

On the 28th of June, 1862, about 9 o'clock, p.m., I was called to see Mrs. P——, a Norwegian, aged 32. She complained of a "lump" in the throat, a slight sick headache, a creeping sensation over all her body, but especially along the spine,—the latter being always in an upward direction. Her countenance was pallid, her eyes somewhat glaring and restless,—the pupils being dilated, her tongue was lightly coated white, except along the edges and tip, which wore a purplish appearance. The temperature of the skin was little above the normal standard, though the pulse was beating at the rate of 100 per minute. Upon her upper lip and forehead were the remains of what appeared to have been small lacerated wounds, but not exhibiting the least sign of pustulation.

As it was rather dark, I opened a window at the head of the bed on which she was lying, for the purpose of admitting a little more light, and the following instant I perceived some spasmodic action in the features; she at the same time raised herself up, and very abruptly closed the window, saying it made her feel cold—although at this time there was scarcely a breath of air to be felt.

Upon this I expected Hydrophobia to be present; and, with the view of testing the correctness of my suspicion, I took a little pulv-Doveri from my pocket-case, mixed it with an ounce of water, and requested her to swallow it. After some little hesitation, she carried it to her lips and withdrew it again; but, upon my urging her to do so, she gulped rather than drank it down, and, with a slightly-perceptible shudder, set down the cup.

I then, in company with her husband, left the room, promising to send her something that would relieve her, and she seemed satisfied. When we were out of her hearing, I asked her husband if she had ever been bitten by a dog, and he replied that she had, under the following circumstances:—

They had recently moved from another portion of the city, and a pet dog which they kept had gone back to the premises they had vacated. The husband set out and brought it back, leaving injunctions with his wife, as he locked the dog in a shed, to keep him there a few days until he became reconciled to the change. (I ought here to remark, however, that, in capturing the dog, he was also bitten by it upon the hand—for which he beat him, and was bitten a second time.) Mrs. P——, a few hours after the dog had been locked up, opened the shed door and commenced scolding him for his truancy; in an instant he sprang upon her, and, after tearing her dress into tatters, bit her in the face and upon the forehead, and rushed from the house, and was not afterwards seen by them.

On receiving the account, I felt *assured* that I had a case of true Hydrophobia to deal with. My next thoughts were—what shall I or can I do?

I prescribed tincture valerianæ, alternated with hydrarg. chlor. mit. and morph. sulph. every three hours, with a view to giving some relief to the patient, and gaining time until I could consult authorities on the subject.

The following morning I called and found my patient had passed a restless night, and seemed rather worse. She complained sorely of thirst, and said that, when she attempted to drink, a lump would rise in her throat and almost choke her. I requested her to drink whilst I was there, so that I might witness its effect upon her. She took a mouthful of water, threw the cup away from her, and grasped her throat violently with one hand, whilst she pressed the other upon her breast, trembling extremely at the same time.

I called Prof. Byford into consultation, and we agreed to continue the opiate in larger doses, ceasing the administration of the valerianæ, and watch for the result of the hydrarg., hoping that if we could get up sufficient alterative action, we might possibly find the case more tractable. We were doomed to disappointment, however; for it appeared to exert no further influence than to produce a few small discharges from the bowels.

With Prof. BYFORD'S concurrence, I proposed to administer chloroform by inhalation—as Dunglison, in his "New Remedies," relates instances where it is reported to have effected cures; but the patient had such a dread of it, on account of its somniferous influence, she begged us to try something instead, first. We then administered P. opii gr. j, every two hours. Up to this period—the end of the second day—she was unable to eat, although there was apparently nothing to prevent it except occasional spasms of a tetanoid character.

On the third day, I found all the symptoms of a more grave character. Pulse extremely small and irregular, but, on the whole, more accelerated than during the two previous days. She feared being choked to death. Temperature of surface, alternately high and low; features cadaverous, and at times covered with clammy perspiration; eyes surrounded by a dark areola; pupils alternately contracted and dilated; hunger and thirst intense, but accompanied by inability to swallow anything more than about a drachm of fluid at once. Efforts to take more than this amount, produced horrible contraction of the facial muscles; and the platysma myoides particularly, and the extraordinary portion, would be sure to be ejected through the mouth and nostrils.

She at this time evinced great exhaustion. Her manner of breathing now commenced to be very laborious and irregular. The act of expiration was accompanied by a sort of stertor, resembling, somewhat, the cough of a person troubled with laryngitis, the only difference being that it was harsher, and made with much greater effort. This, I suppose, is what is *popularly* termed the "barking," in such cases.

At this stage I informed her of the probable termination of her case, and urged her to inhale the chloroform, but she again refused: this time, however, with the understanding that if she were "not better to-morrow," she would permit me to do what I might consider best. During this day, she obtained partial relief by the exhibition of P. opii gr. ji. every hour, during the earlier, and every half hour during the latter part of it.

Through all this time, she was rational and patient. Her husband's fears had caused him to remove her two children from the house, but she requested that they might remain at the window, so that she might see them at least. She could not now bear the window or door open,—the slightest breath of air being productive of frightful contortions of the whole body.

At 10 o'clock, p.m., of the third day, she complained of suffocation, and begged her husband to go to the door with her "to get a breath of air." He did so; and, after remaining about 30 seconds, she returned into the house and went to bed. About 11 o'clock she called for water, and he gave her about half a teacupful; she readily drank nearly the half of it, and, saying "It is hard so, but I must die," she sighed deeply, shuddered and died, thus preventing a trial of the chloroform.

Some of the circumstances attending this case are remarkable, as they conflict the written authorities; for instance:—Instead of a red, animated countenance, we had an extremely anxious, careworn and pallid one. Again; the horror, said to exhibit itself on the sight of fluids, did not exist in this case till after the trial to drink; and drinks were generally, in this case, received with fervor. Again; there was no grinding of the teeth, which we are taught to look for as a prominent symptom; and the tumors spoken of by Cooper, as existing under the tongue, did not show themselves; and, last of all, we have positive knowledge that the disease set in on the eighth day after being bitten; and, where the biting was done, she had no pain or itching.

